# Supplementary material for: Challenges and recommendations to improve the installability and archival stability of omics computational tools
Source: PLoS Biol. 2019 Jun 20;17(6):e3000333. doi: 10.1371/journal.pbio.3000333 (PMC6605654; doi:10.1371/journal.pbio.3000333)
Supplement: S1 Text — (PDF) [file pbio.3000333.s001.pdf]

Supplemental Note 1. An example of the 'Quick Start'

1. Download the tool using: git clone https://github.com/x/software.tool.git
2. Install tool using: cd software.tool; ./install.sh
3. Run the tool for the example dataset (distributed with the tool): ./software.tool example.dataset
