# Supplementary material for: Challenges and recommendations to improve the installability and archival stability of omics computational tools
Source: PLoS Biol. 2019 Jun 20;17(6):e3000333. doi: 10.1371/journal.pbio.3000333 (PMC6605654; doi:10.1371/journal.pbio.3000333)
Supplement: S2 Text — (PDF) [file pbio.3000333.s002.pdf]

Supplementary Note 2. Automatic verification of software installability

Software quality, including installability, is typically not thoroughly tested in the formal peer review process, and relying on reviewer feedback can be problematic, as the reviewers may lack the computational skills and time to verify the tools. It is possible to automate the assessment process when software guarantees access to (i) the software binaries or source code; (ii) a script that installs the software in a given Linux environment; (iii) a small example dataset and its expected output; and (iv) a script to perform the analysis on the dataset from *(iii)*.

To provide an automated and openly verifiable certification that a tool is usable, we suggest a model of a server that uses public badges to endorse the installability of a software tool. The server will issue a certificate to the software author, which indicates that the proposed software passed an 'Automatic Installation Test.' The installation process, in this case, includes a testing phase that ensures the installation can be successful. Authors of computational tools who submit their software tool to our badge server, alongside an installation script and an example dataset, will receive a badge of confirmation which certifies that the software tool was successfully installed in a third-party environment. Using a Secure Hash Algorithm [1], each generated badge would be unique to each version of the software, installation script, test dataset, and operating system used by the server.

To validate a badge, the server will use a private cryptographic key to publicly sign the badge. Public badge testing provides a strong endorsement of the tool installability up to the current highest standards in the industry, as only the same software version, installation script,

and test dataset will confirm the authenticity of the badge and its public signature. A public badge platform will provide a mechanism for researchers and editors of journals in computational biology to verify the installability of a tool in under five minutes through the confirmation of the server's signature. Badges inform the user *a priori* if and under what conditions the software is installable, potentially reducing for each user a significant amount of time that otherwise would be required to test software and attempt installing software that is ultimately uninstallable.

In addition to guaranteeing that a software tool can be successfully installed in a standardized environment, the badge also reflects which specific Linux system was used during the test installation. (Linux-based systems are the most commonly used operating systems in the field of computational biology.) Furthermore, the badge server does not assume an open source software and can be generated based on the source code or binary files.

The server creates an instance of a Linux virtual machine and runs the installation script and test protocol submitted. If the installation completes without errors, and the test dataset provides the expected result, a badge is created that certifies the installability of the tool under the tested conditions. The badge consists of a unique summary generated by the Secure Hash Algorithm 3 (SHA-3) [1], of the items submitted by authors. The server then uses a private cryptographic key to publicly sign this summary. There is a small probability that the hashed summary of two different objects created by SHA-3 could be identical (also known as collision); however, this method is the current technological standard of unique badge creation and is broadly accepted by all industries. Using the server's public key and the hashed version of the

software, any user can authenticate the signature and prove that the server was indeed able to install the tool without manual intervention.

References

1. Dworkin MJ. SHA-3 Standard: Permutation-Based Hash and Extendable-Output Functions [Internet]. 2015 Aug. Report No.: Federal Inf. Process. Stds. (NIST FIPS) - 202. Available: https://dx.doi.org/10.6028/NIST.FIPS.202
