# Supplementary material for: Challenges and recommendations to improve the installability and archival stability of omics computational tools
Source: PLoS Biol. 2019 Jun 20;17(6):e3000333. doi: 10.1371/journal.pbio.3000333 (PMC6605654; doi:10.1371/journal.pbio.3000333)
Supplement: S3 Text — (PDF) [file pbio.3000333.s003.pdf]

Supplementary Note 3. List of bioinformatics tools with name Prism.

- https://www.ncbi.nlm.nih.gov/pubmed/22851530 (Structural Variance)
- https://academic.oup.com/nar/article/43/20/9645/1394603 (Metabolomics)
- https://www.ncbi.nlm.nih.gov/pubmed/21068001 (Viral Genomics)
- http://honig.c2b2.columbia.edu/prism/ (Protein Structure Analysis)
  https://www.ncbi.nlm.nih.gov/pubmed/15991339 (Protein Structure)
- https://www.bits.vib.be/software-overview/graphpad-prism (Statistics and visualization)
