# Supplementary figures and images for: Challenges and recommendations to improve the installability and archival stability of omics computational tools
Source: PLoS Biol. 2019 Jun 20;17(6):e3000333. doi: 10.1371/journal.pbio.3000333 (PMC6605654; doi:10.1371/journal.pbio.3000333)

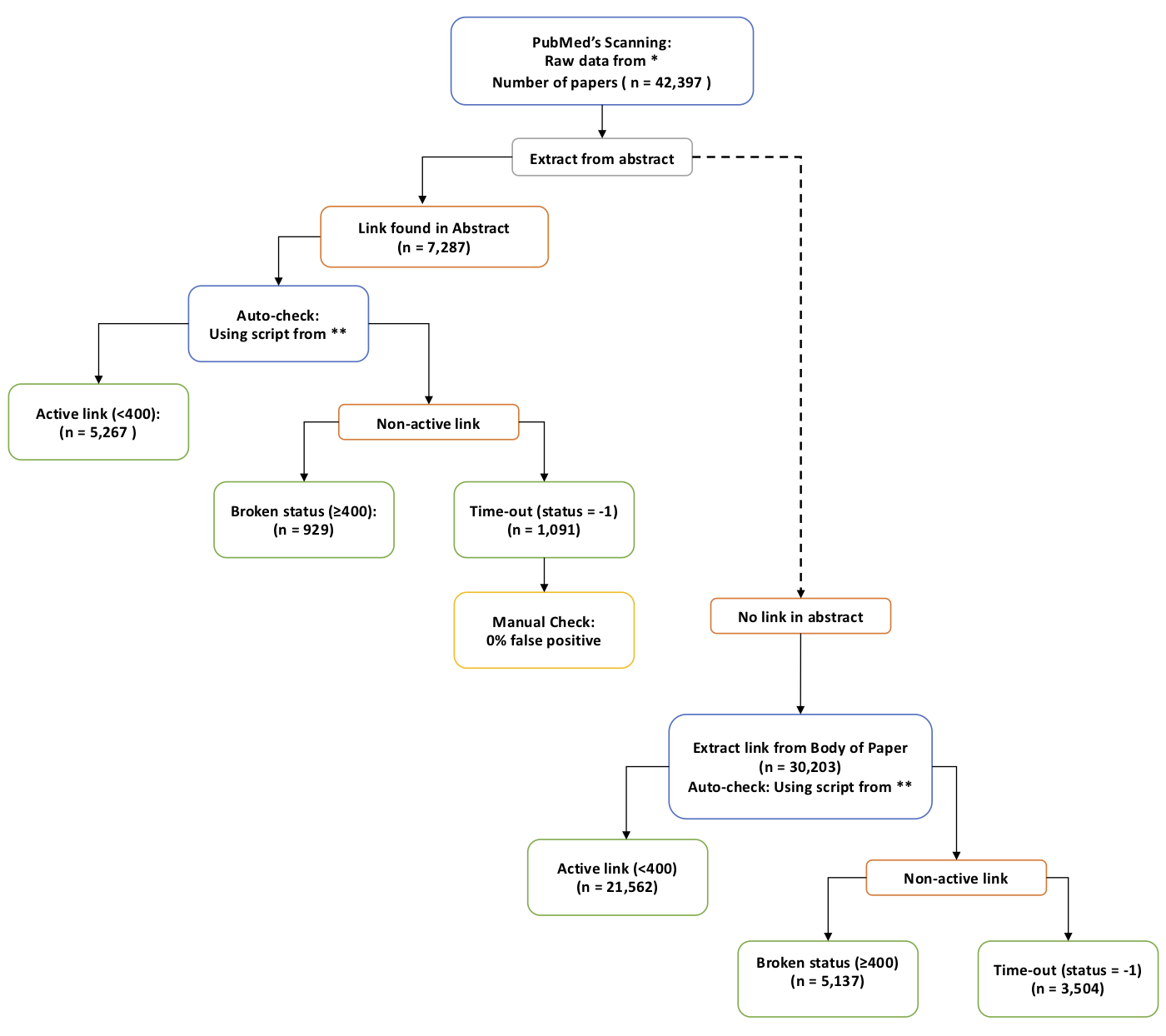

Supplement: S1 Fig — Numbers are provided for illustrative purposes and correspond to the link presented in the abstracts of the published papers considered in this study. (TIFF) [file pbio.3000333.s007.tiff]

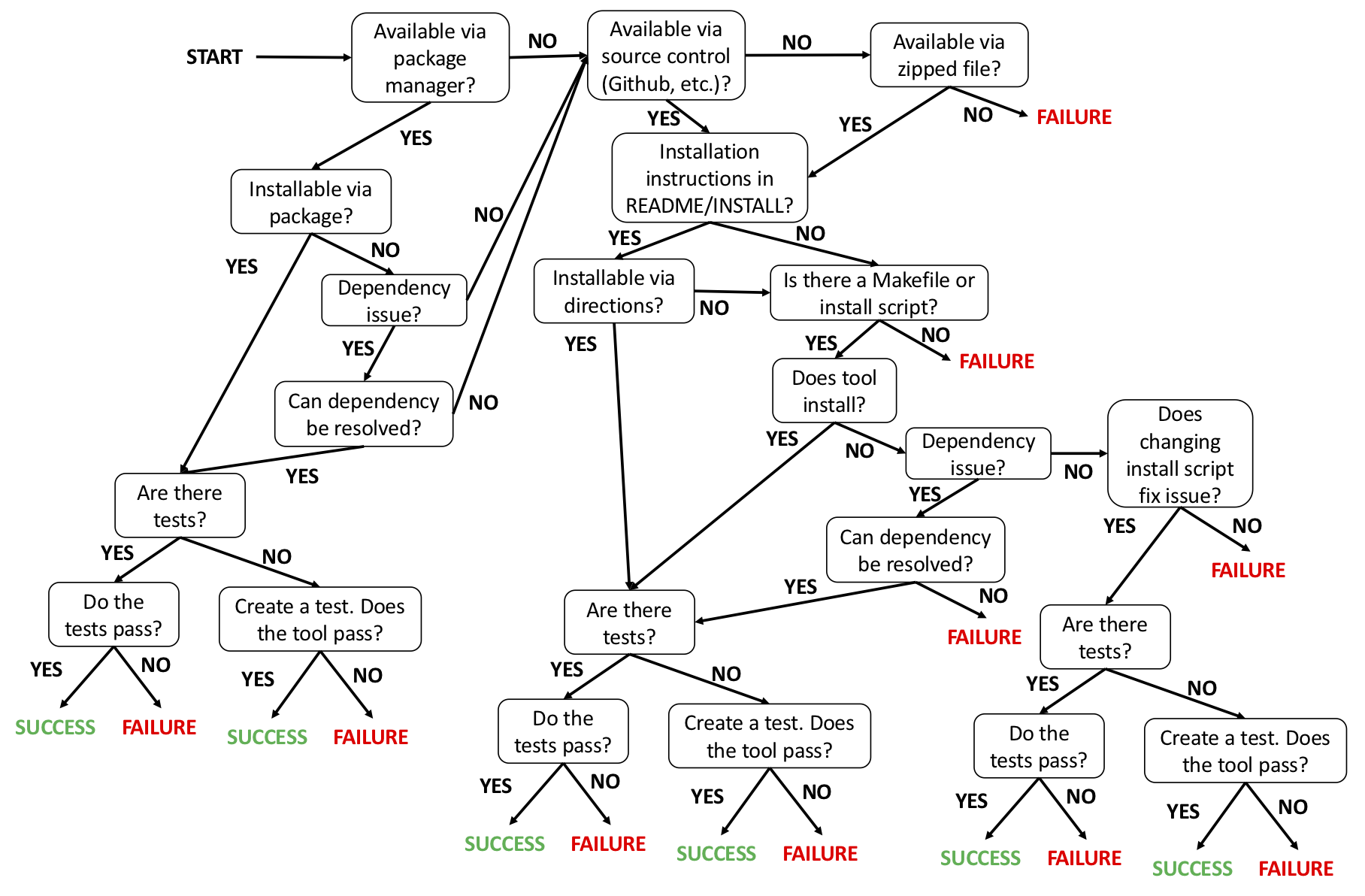

Supplement: S2 Fig — (TIFF) [file pbio.3000333.s008.tiff]

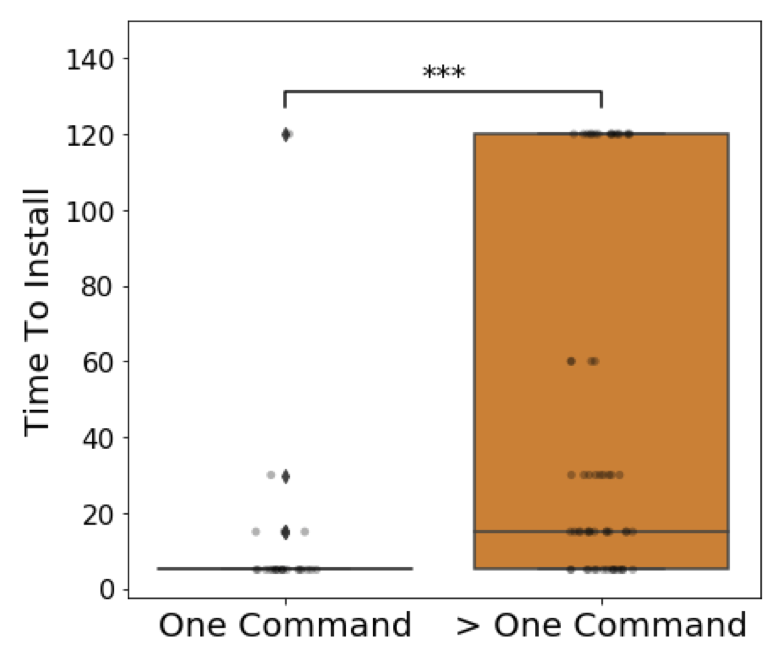

Supplement: S3 Fig — (TIFF) [file pbio.3000333.s009.tiff]
